# Potential Clinical Applications of Exosomal Circular RNAs: More than Diagnosis

**DOI:** 10.3389/fmolb.2021.769832

**Published:** 2021-11-24

**Authors:** Kearabetsoe Matseliso Molibeli, Rong Hu, Yuze Liu, Dehui Xiong, Lijun Tang

**Affiliations:** ^1^ School of Life Science, Central South University, Changsha, China; ^2^ Xiangya School of Medicine, Central South University, Changsha, China

**Keywords:** exosomes, exosomal circRNA, biomarker, miRNA sponge, diagnosis, therapeutic target

## Abstract

Exosomes are small vesicles derived from cells used as cell-to-cell communication goods in numerous diseases including tumorigenesis, neurological diseases, cardiovascular diseases and other diseases. Circular RNAs (circRNAs) are an innovative constituent of non-coding endogenous RNAs generated through backsplicing, catalyzed by RNA polymerase Ⅱ. These non-coding RNAs have been suggested to control gene expression through miRNA sponging, RNA-binding protein regulation and translational capabilities. Genome-wide RNA sequence analyses observed that circRNAs were stably improved in exosomes in association to parental cells. Little attention has been dedicated to exosomal circRNAs (exo-circRNAs). However, research has demonstrated that exo-circRNAs may have important regulatory functions because of their stability in cells and within exosomes. If well understood, the precise roles and mechanisms of exo-circRNAs might surge the impending clinical applications of these molecules as markers in the identification, prediction and treatment of various diseases. In this review, we outline recent findings regarding exo-circRNAs which includes their functions and highlights their potential applications and therapeutic targets in human diseases.

## Introduction

Exosomes are nano-sized (30–150 nm) extracellular vesicles that are released by majority of distinctive types of cells and distributed in body fluids, such as cerebrospinal fluid, synovial fluid, saliva, cerebrospinal fluid, urine, breast milk, blood and so on. Over the past 30 years there has been an increased attentiveness in exosomal research due to their unique functions as intercellular messengers and involvement in varied biological processes including, but not limited to; cell proliferation, cell migration, cell differentiation, angiogenesis, antigen presentation, and immune responses (Dai et al., 2020). Several cellular steps commence the biogenesis of exosomes and these components are constituently generated from late endosomes generated through inward budding of multivesicular body (MVB) membrane. This inward budding of the membrane leads to the formation of intraluminal vesicles (ILVs) in MVBs (Théry et al., 2018). In this process certain proteins are incorporated into the invaginating membrane, while the cytosolic components are engulfed and enclosed within the ILVs. Evidence has revealed that the formation of ILVs requires the function of a protein machinery endosomal sorting complex required for transport (ESCRT) function that is composed of four separate protein ESCRTs (0 through III) working cooperatively to facilitate MVB formation, vesicle budding, and protein cargo sorting (Agromayor et al., 2012; Zhang et al., 2019). Most ILVs are eventually released into the extracellular space upon fusion with the plasma membrane, which are then referred to as “exosomes” (Mashouri et al., 2019). Initially, the primary role of exosomes was thought to be cellular waste removal and because of their presence in almost all biological fluids, exosomes have progressed to become such useful biomarkers due to their key function in cell to cell communication and thus messengers of health and disease (Vidal, 2019). Recently the specific applications of exosomes in various diseases is growing rapidly. Another review focusing on functional mechanisms of exosomes in the articular microenvironment in knee osteoarthritis documented the therapeutic effects of mesenchymal stem cell (MSC)-derived exosomes. Injection of exosomes derived from MSCs into the joint cavity will change the molecular composition and improve the microenvironment of the joint cavity as exosomes derived from MSCs can inhibit inflammation and apoptosis of chondrocytes (Li et al., 2020). Furthermore, therapeutic potential of exosomes in cardiac tissue engineering and coronary artery disease (CAD) has been recorded. MSC-derived exosomes have been mentioned to possess the potential to regenerate the injured cardiac tissue by preventing apoptosis and promoting the angiogenesis to restore the blood flow (Thankam and Agrawal, 2020). As exosomal content is extensive and consists of various growth factors, proteins, lipids, DNA, and non-coding RNAs from the cells that release them, once released into the extracellular matrix they are taken up by distant cells where they eventually provoke responses in target cells through several paths including fusion with plasma membrane, endocytosis, and binding on the cell surface (Raposo and Stoorvogel, 2013; Zhang et al., 2018). Results from “cell counting kit 8 (CCK8), wound healing and transwell assays” indicated that exosomes significantly increased colorectal cancer (CRC) cell proliferation and markedly promoted the migration and invasion of CRC cells compared to that in control groups (Shang et al., 2020). The increasing interest in exosomes in recent years is due to their important purposes in health and diseases as well as their potential clinical application in therapy and diagnosis. Thus, in addition to their ability to cross the blood brain barrier, exosomes have the potential to aid in disease diagnosis and targeted drug delivery acting as delivering therapeutic agents to a desired target (Saeedi et al., 2019; Wang et al., 2019; Huda et al., 2021; Massey et al., 2021). Various methods have been developed and implemented for the isolation of exosomes from biological fluids that include centrifugation, size exclusion chromatography, filtration, polymer-based precipitation, immunological separation and isolation by sieving. Selection differs depending on the biological fluid and amongst these methods, differential centrifugation is one of the most commonly used technique (Alzhrani et al., 2021). The physical properties of exosomes are characterized according to their size, shape, surface charge, density, and porosity in order to determine their biological interactions. Thus, nanoparticle tracking analysis (NTA), dynamic light scattering (DLS), resistive pulse sensing, atomic force microscopy (AFM), transmission electron microscope (TEM) and flow cytometry have been used for the determination of exosomal characteristics (Gurunathan et al., 2019).

### Circular RNA Characteristics and Functions

**FIGURE 1:**
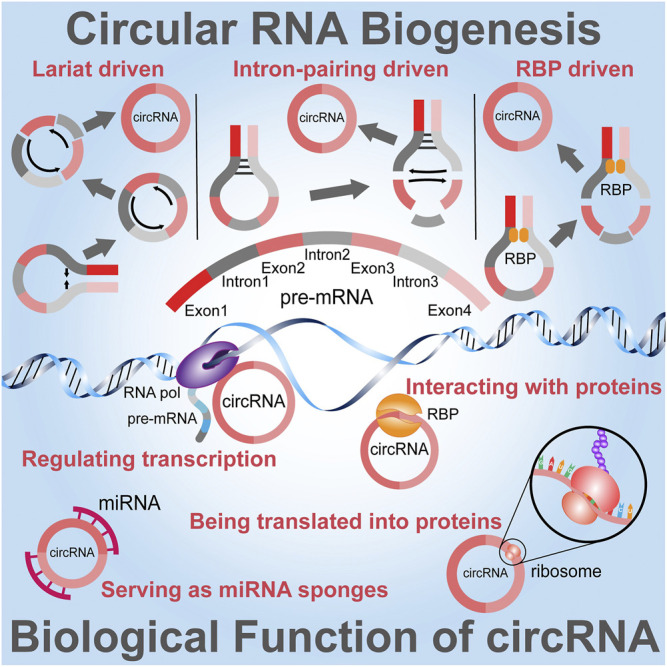
Overview of the Biogenesis and Functions of circRNA. Three models are used to illuminate the possible formation of circRNAs. Lariat driven circularization model contains exon 2 and exon 3 produced from exon skipping. The lariat subsequently undergoes internal splicing and circRNAs are generated by removing introns 1, 2, and 3. Intron pairing driven circularization or direct backsplicing introns are paired by base pairing to form a circular structure. In RNA binding protein (RBP) driven model, the RBP between intron 1 and intron 3 mediates this circularization. The important biological functions of circRNAs include transcription regulation, protein interaction and being translated into proteins and interacting with miRNAs through miRNA sponging.

The size of a spliced circRNA molecule can range from smaller than 100 nt to longer than 4 kb and are divided into 4 categories; exonic circRNAs (ecicRNAs), intronic circRNAs, exon-intron circRNAs (EIcircRNAs), and intergenic circRNAs (Szabo and Salzman, 2016). Evidence has revealed that circRNAs display various functions by behaving as gene expression modulators (Jiao et al., 2021), serving as miRNA sponges (Kumar et al., 2017), interacting with proteins (Mo et al., 2021), regulating transcription (Verduci et al., 2021) and translated into proteins (Fontemaggi et al., 2021) (Figure 1). Since exosomes and their biologically active cargoes may offer prognostic information in a range of diseases, as one of their cargo components circRNAs have become a research hotspot in recent years because of their close relation with the development of diseases (Li et al., 2021). These molecules are characterized by a covalently closed loop configuration created through a back-splicing occasion whereby a 3′-5′ phosphodiester bond is formed (Deng et al., 2019). To further explore the mechanism of circRNAs; circRNA-miRNA system analysis is normally constructed to reveal the molecular regulatory networks. For instance, circLONP2 directly interacted with primary miRNA-17 (pri-miR-17) and promoted the processing of this miRNA (Zhu et al., 2020). Furthermore, knowledge of exo-circRNA-miRNA interaction and their mechanisms that influence gene expression has currently been recorded, exosomal circRNA_100284 originating from arsenite-transformed L-02 cells persuaded cell cycle acceleration and encouraged proliferation by sponging miRNA-217 (Dai et al., 2018) and additionally, exosomal circPDE8A promoted the EMT in recipient cells via activating the MACC/MET/ERK or AKT pathway (Li et al., 2018). Since genome-wide RNA-seq analyses revealed that circRNAs were enriched in exosomes compared to parental cells (Shang et al., 2020); detected the presence of circRNA in exosomes using transmission electron microscope (TEM) and nanoparticle tracking analysis (NTA) method. Exo-circRNAs can also be acquired by adjacent or distant cells and have a prospective to affect various features of physiological and pathological conditions of the recipient cells. It is to be noted that the expression profile of exo-circRNAs in recipient cells is dissimilar from that of donor cells, suggesting that circRNAs are successfully loaded from donor cells into exosomes then transported to recipient cells (Deng et al., 2020; Guo et al., 2021). Although the biological functions of exo-circRNAs remain partly interpreted, a better understanding can render innovative discernments into the pathogenesis and treatment of various diseases, it could allow future studies to identify new prospective exosome-based disease biomarkers. The mechanisms or potential effects of differentially expressed exo-circRNAs should be further analyzed to broaden the spectrum of their clinical applications. In this review, we expand on how exo-circRNAs are engaged in different diseases, the working mechanisms by which they play part in human diseases and their applications.

### Exosomal Circular RNAs in Tumorigenesis

Recently, circRNAs have been described to be stable and highly enriched in exosomes (Li Y. et al., 2015; Michaelidou et al., 2020) and have found that abnormally expressed exo-circRNAs may be associated with the development and progression of malignancies. For example, tumor cells release exosomal circ-PDE8A that in turn facilitates invasive advances in a miR-338-MET transcriptional regulator MACC1-MET protooncogene receptor tyrosine pathway-dependent manner in pancreatic cancer cells, showing that the invasiveness of tumor is enhanced by the transfer of circ-PDE8A facilitated by exosomes (Li Z. H. et al., 2018). Another study reported that exosomal circRNA_100284 enhanced cell division cycle and permitted proliferation through sponging miR-217. Over-expression of circRNA_100284 increased the formation, invasion and migration of tumor colonies through stimulation of the downstream signaling pathway and an increase of the expression of EZH2 and cyclin-D1 in human hepatic cells. Additionally, this circRNA also has an impact on the benign transformation of cells (Dai et al., 2018). circHIPK3 has been recorded to regulate cell proliferation, differentiation, and migration, and consequently playing a key role in disease processes (Zhou J. et al., 2021). These exosomes based circRNAs have showed key factors in cancer development and progression, and thus have aroused more and more attention. In distinction to tumor-derived exo-circRNAs stimulating proliferation in recipient cells, exo-circRNAs derived from normal cells prevent proliferation in these cells.

### Hepatocellular Cancer

Exosomes function in the development of HCC and since cell-to-cell communication is important between cancer cells and their microenvironment, it is additionally responsible for disease progression. The exosomal cargoes could serve as delivery systems for the prediction and diagnosis of HCC (Li L. M. et al., 2019). Exosomes that are derived from metastatic HCC cell lines comprise a great number of pro-tumorigenic RNAs and proteins with circRNAs identified to be important regulators in human cancer; the pro-invasive role of exosomal circRNA-100338 in HCC metastasis was explored and its high expression in metastatic HCC cells and their secreted exosomes was exhibited (Huang et al., 2020). Thus, the metastatic ability of HCC cells could be enhanced by transferring exo-circRNAs. Following this previous research on the differential expression of exo-circRNAs, studies in HCC have characterized exo-circRNAs originating from different types of donor cells and revealed their effects in recipient cells on cell growth, metastasis, and drug resistance; indicating material exchange between donor and receipt cells. For instance, circ-0051443 is transferred from normal cells to HCC cells via exosomes and suppresses the malignant biological behaviors by promoting cell apoptosis, arresting the cell cycle and also decreasing the weight and volume of the xenograft tumors in nude mice via *BAK1* upregulation in these tumors (Chen et al., 2020). Exosomal circ-ZNF652 could be transported to HCC cells and its silencing inhibited HCC cell proliferation, migration, invasion and glycolysis. Moreover, circZNF652 knockdown blocked tumor growth *in vivo* (Li et al., 2020). Evidence has shown that exo-circRNAs contribute to HCC progression through modulating the expression and function of their miRNA target genes. UHRF1-derived circRNA expression profiles in human HCC tissues were analyzed in non-tumor tissues and HCC derived exosomes. HCC-derived exosomal circUHRF1 was found to upregulate the expression of the miR-449c-5p target gene TIM-3 in NK cells by combining with and degrading miR-449c5p, leading to the promotion of immune evasion and resistance to anti-PD1 immunotherapy in HCC (Zhang et al., 2020). Results showed in oral squamous cell carcinoma (OSCC) cells and tissue, circUHRF1 was markedly upregulated and closely correlated with poor prognosis of OSCC patients. Functionally, circUHRF1 promoted the proliferation, migration, invasion, and epithelial mesenchymal transition (EMT) *in vitro* and promoted tumor growth *in vivo*. Mechanically, circUHRF1 acted as miR-526b-5p sponge, so positively controlling c-Myc protein. Through western blot analysis it was validated that miR-526b-5p mimics transfection reduced c-Myc protein and its inhibitor transfection increased c-Myc protein denoting that this protein functions as the target of circUHRF1/miR-526b-5p (Zhao et al., 2020). Therefore, expression patterns of some exo-circRNAs are multifaceted and may demonstrate diverse functions among different cell types and tissues in different diseases. Because exosomal circUHRF1 was identified in HCC derived exosomes, it was further explored whether it was present in HCC patient plasma. After tumor resection plasma exosomal circUHRF1 levels were reduced and increased in patients with tumor relapse, indicating that this circRNA was mainly produced by HCC cells. Exosomal circUHRF1 levels were markedly increased in the plasma of patients with signs of immune evasion mechanisms, which had a decreased NK cell proportion in peripheral circulation. Plasma exosomal circUHRF1 could then serve as a critical molecular determinant of NK cell-related immune evasion (Zhang et al., 2020).

### Non-Small Cell Lung Cancer

Non-small cell lung cancer (NSCLC) has been reported to be the leading cause of deaths related to cancer worldwide and accounts for the most diagnosed type of lung cancer (Duma et al., 2019). Information on the purposes and mechanisms by which circRNAs are involved in lung cancer is limited and more exploration is needed. However, circRNAs have been reported to participate in the regulation of protein expressions (Du et al., 2017). A research reported circSATB2 as a participant in the progression of NSCLC through positively regulating fascin homolog 1, actin-bundling protein 1 (FSCN1) expression via miR-326 and was differentially expressed in lung cancer tissue and serumal exosomes (Zhang et al., 2020). Furthermore, in NSCLC serum-derived exosomes, tumor tissues and cells hsa_circ_0014235 expression was notably higher (Wu et al., 2020). In a recent study, circRNA_102481 silencing transferred by exosomes could impede EGFR-TKIs resistance, cell proliferation and promote cell death (Yang et al., 2021). It is therefore essential to determine molecular mechanisms behind NSCLC tumorigenesis. CircRNA-002178 could also be discovered in lung adenocarcinoma (LUAD) patient plasma exosomes, it was also found to be delivered into CD8^+^ T cells to induce PD1 expression via exosomes (Wang et al., 2020). Other types of circRNAs found in exosomes including exosomal circ_0047921, circ_0056285 and circ_0007761 are promising predictors for NSCLC identification in the Chinese population (Xian et al., 2020). ROC curve analysis discovered that increased expressions of serum exosomal circ-MEMO1 may be a valuable diagnostic marker for NSCLC because circ-MEMO1 was upregulated in NSCLC. Such high expression predicted poor prognosis in NSCLC patients (Ding et al., 2020). Moreover, higher expression of circ_0067934 led to a significantly poorer survival indicating that circ_0067934 had an independent influence on the poor prognosis of patients with NSCLC (Wang J. and Li, 2018). It is to be noted that the purpose that circRNAs play and their mechanisms in lung cancer needs more exploration (Zhang et al., 2020).

### Colorectal Cancer

Tumor microenvironment has been proposed to be associated with colorectal cancer (CRC) development. Cancer-associated fibroblasts (CAFs) are considered to be one of the key stromal cells in tumors (Gu et al., 2020). The prognosis of CRC has never been satisfactory however; targeted therapy is an optional method that has effectively elongated overall survival for CRC patients. Exo-circRNAs have been recognized in CRC to participate in invasion, proliferation, metastasis and apoptosis (Han et al., 2020). A new CRC-derived exo-circRNA, circPACRGL, was uncovered to be considerably upregulated in CRC cells after the addition of tumor-derived exosomes. As a result, circPACRGL promoted CRC cell proliferation, migration and invasion (Shang et al., 2020). A study aiming at examining the probable clinical use of serum exo-circRNAs in diagnosing CRC was shown. Validation evaluation discovered that serum exosomal circ-PNN (hsa_circ_0101802) levels were considerably upregulated in CRC cases in comparison healthy control groups (Xie et al., 2020). Exosomal circ_0067835 was found to be upregulated in CRC patients’ serum after radiotherapy and its knockdown inhibited cell proliferation, cell cycle progression, and further enhanced cell apoptosis and radio sensitivity *in vitro* (Wang et al., 2021). Cell proliferation, invasion and apoptosis together with exosomal circSLC7A6 and CXCR5 expression levels were analyzed in another study. It was found that circSLC7A6 acted as a promoter for CRC cell proliferation and invasion, whereas as an inhibitor for apoptosis. Their data showed that matrine inhibited CRC tumorigenesis by hindering the release of exosomal circSLC7A6 from CAFs (Gu et al., 2020). Data in CRC research also presented that CRC-derived exosomal circPACRGL stimulated CRC proliferation and metastasis, together with the differentiation of N1-N2 neutrophils via controlling miR-142-3p/miR-506-3p-TGF-β1 axis (Shang et al., 2020) and an exo-circRNA derivative, exosomal circ-133, from hypoxic cells transferred into normoxic cells and stimulated colorectal cancer metastasis through acting on miR-133a/GEF-H1/RhoA axis (Yang et al., 2020).

### Ovarian Cancer

The current diagnostic and therapeutic state of OC includes surgery and chemotherapy, and due to shortage of effectual primary detection screening tests, the prognosis remains poor (Wang et al., 2020). Therefore, several findings have exhibited that circRNAs are differentially articulated in various OC tissues which proposes the significance of circRNAs in the progression of OC. For instance, Cdr1as was downregulated in cisplatin-resistant patient tissues and cell lines and its overexpression inhibited the proliferation of cells and promoted cisplatin-induced cell death in ovarian cancer cells (Zhao et al., 2019). In another study, the expression level of circPUM1 was expressively higher in OC tissues than in normal ovarian tissues. When compared with the control group, circPUM1 over-expression promoted CAOV3 cell growth, constrained apoptosis, and intensified migration, invasion, and contributed to metastasis of cancer in the form of cancer originated exosomes (Guan et al., 2019). The role of serum exosomal circular forkhead box protein P1 (circFoxp1) on survival outcome and cisplatin (DDP) resistance in patients with epithelial ovarian cancer (EOC) was investigated. Circulating exosomal circFoxp1 was found to be significantly increased in patients with EOC, especially in DDP-resistant EOC patients, becoming an independent factor in predicting survival and disease relapse in patients with EOC. Overexpression of this exo-circRNA promoted cell proliferation and conferred DDP resistance, while knockdown of circFoxp1 inhibited cell proliferation and enhanced DDP sensitivity both *in vitro* and *in vivo* (Luo and Gui, 2020). Additionally, circ-0001068 was discovered to be significantly higher in serum exosomes from OC patients compared to healthy volunteers. Circ-0001068 was also transported into T cells and persuaded PD1 manifestation by operating as a competing endogenous RNA (ceRNA) for miR-28-5p via exosomes (Wang et al., 2020). Although surgery merged with chemotherapy has been implemented as the foremost treatment for OC, the reoccurrence rate of OC remains high therefore, early detection is peculiarly important to OC.

### Gastric Cancer

Gastric cancer is a foremost source of death in modern-day society (Wei et al., 2020). The expression features of circRNAs in plasma exosomes have in plasma exosomes have specific characteristics in GC; for example, ciRS-133 was linked with the browning of white adipose tissue (WAT) in GC patients (Zhang et al., 2019). In order to broaden perspective on the roles of exosomes in invasion and metastasis in GC, (Yan et al., 2017), concentrated on recent findings on GC exosomes by summarizing their functions, as well as their clinical application outlook. In GC tissues and serum circSHKBP1 (hsa_circ_0000936) levels were elevated and connected to advanced TNM stage and poor survival, promoted angiogenesis, GC cell proliferation, migration an invasion both *in vitro* and *in vivo*, while its inhibition functioned oppositely (Xie M. Y. et al. (2020)). Moreover, exosomal circRanGAP1 showed potential in gastric cancer progression; the plasma exosomes derived from patients caused enhanced migration and invasion of gastric cancer cells (Luo and Gui, 2020). Furthermore, the overexpression of circ-ITCH inhibited the proliferation, migration, invasion as well as epithelial mesenchymal transition (EMT) of GC cells, whereas its knockdown emerged to exert an opposite outcome (Wang et al., 2021). Using a co-culture system, the transmission of circNRIP1 was traced through exosomal communication. It was documented that circNRIP1 can be transferred by exosomes between GC cells, and promote tumour metastasis *in vivo* (Zhang. et al., 2019).

### Prostate Cancer

Prostate cancer is one of the most common aggressive tumours in males. Although improvements have already been implemented and obtained in screening, diagnosing, and in the treatment of PC, there is a decrease in the mortality and low overall survival rates (Li et al., 2020). A large amount of evidence indicates that circRNAs in exosomes are crucial in the invasion and metastasis of PC (Gao et al., 2021). In a study to identify differentially expressed circRNAs in PC tissues, circ_0088233 expression level was discovered to be upregulated in PC tissues in association to adjacent normal tissues (Deng Z. H. et al., 2020). In another study, the knockdown of circ_0061140 inhibited the proliferative potential of PC cells (Wang et al., 2021). On that note, exo-circRNAs have been described to have an influence in the incidence and advancement of PC. However, the mechanisms of the initiation and progression of PC together with the purpose of exo-circRNAs from PC patients are not fully clarified thus; research is being conducted to investigate their use in PC. For example, a recent report found that circ_0044516 was shown to be highly expressed in exosomes from PC cases and cell lines. Additional exploration established that circ_0044516 downregulation prohibited proliferation and metastasis of PC cells. This study thus denoted that circ_0044516 participated in PC cell survival and metastasis, displaying an oncogenic purpose of circ_0044516 in PC (Li T. et al., 2021). Although there are reports on circRNAs in PC that have been pointed out that they may become useful for monitoring proliferation and metastasis; the current state of the mechanisms of exo-circRNAs in PC needs more attention as the research is very limited.

### Exosomal Circular RNAs in Neurological Diseases

Neurological diseases target the nervous system including the brain, spine or nerves and the second leading cause of death (Dumurgier and Tzourio, 2020). Studies have proposed that circRNAs may play fundamental roles in the occasion and development of brain related diseases and therefore may have potential as innovative biomarkers (Borsook, 2012). CircRNAs in Alzheimer’s disease (AD) regulate disease on set and have been reported to be specifically enriched in the nervous system (Hosaka et al., 2019). Recent studies have also shown that a large number of circRNAs exist in the hematopoietic system and participate in neuron development (Floris et al., 2017; D'Ambra et al., 2019). Other studies have presented circRNAs to be vigorously modified during the development of neurons and throughout aging, therefore the risk of being exposed and affected by ND rises intensely with age. To further investigate the relationship amid plasma circRNA_089763 level and post-operative cognitive dysfunction (POCD) in aged individuals after non-cardiac surgery, (Zhou et al., 2020), demonstrated that circRNA_089763 level is higher in the POCD group compared to NPOCD group. At present, few studies have directly assessed the functions and mechanisms of circRNAs in NDs; rather, most are only related to circRNAs functions as miRNA regulators. Since circRNAs have the capacity to act as miRNA sponges in order to control gene expression (Zhuo et al., 2020), exosomal miRNAs have been identified in NDs and their analysis has also been proposed for the diagnosis of several NDs. Treatment of miR-124-3p upregulated exosomes hindered neuronal inflammation in scratch-injured neurons and contributed to neurite outgrowth (Huang et al., 2018). In comparison, another study focusing on the purpose microglial exosomal miR-124-3p (EX0-124) play on controlling post-traumatic neurodegeneration proved this exosomal miRNA to be significantly changed in acute, sub-acute, and chronic phases after repetitive mild traumatic brain injury (rmTBI) and *in vitro* experiments showed that upregulated EXO-124 lessened neurodegeneration in repetitive scratch-injured neurons (Ge et al., 2020). As it has been demonstrated that neurons can secrete exosomes (Yu et al., 2020) and exosomes have the ability to transport bioactive molecules across the blood-brain barrier to the blood and then CSF, recommending that circRNAs can travel out of the brain through the aid of exosomes. The pathogenesis of Parkinson’s disease (PD) progresses through intercellular communication between cells that takes place via extracellular vesicles (EVs) (Yu et al., 2020). One publication in Schizophrenic patients was conducted to analyze the alterations of circRNA expression in plasma exosomes. Eight differentially expressed circRNAs were positively identified and shown to encompass binding locations to various miRNAs. These differentially expressed circRNAs played potential roles in metabolic process, stress response, and histone ubiquitination providing understanding on the pathogenesis of schizophrenia at molecular levels (Tan et al., 2021). The research that has already been conducted on exosomal miRNAs can provide direction on the applications of exo-circRNAs for future studies in NDs. The challenge that remains is that the exact overall function of circRNAs in NDs is still unknown because of the limited research available therefore, there is a need for future studies to show their impact (Floris et al., 2017). These characteristics suggest that circRNAs could play important roles in nervous system diseases such as Parkinson’s Disease, Alzheimer’s Disease and others.

### Exosomal Circular RNAs in Cardiovascular Diseases

The connection of exo-circRNAs with CVDs is less studied with very few published researches. The current knowledge of the active roles of circRNAs in the advancement of CVDs is unknown and there is no specific CVD that can be exclusively correlated with circRNAs because of the limited understanding (Altesha et al., 2019). Furthermore, circRNA expression in the peripheral blood of CAD patients was investigated and its relationship with CAD severity determined. The study found that hsa_circ_0124644 may be studied for diagnosing CAD (Zhao et al., 2017). Some studies have suggested that the role of circRNA in the development of CVDs can be entirely associated with miRNA as it significantly regulates every target and the overall function of the mRNA to cause the disease (Kishore et al., 2020). An analysis investigated the expression levels of exo-circRNA in the plasma of CAD patients compared with non-CAD controls and reported hsa_circ_0005540 being significantly associated with CAD with a (*p* < 0:0001) value. This suggested that this specific plasma exo-circRNA can be used in the identification of CAD (Wu et al., 2020). An in-depth research is required in the applications and mechanisms of exo-circRNAs in CVDs.

### Exosomal Circular RNAs in Other Diseases

Presently, circRNAs are viewed as good targets for biological markers because of their stable enrichment in plasma and serum exosomes. For example, in a study that aimed to profile, speculate and probe the functions of circRNAs that are differentially expressed in plasma exosomes of cases with Grave’s Disease and healthy controls, an intonic circRNA hsa_circRNA_000102 was then identified as an upregulated component in plasma exosomes from patients Grave’s Disease (Sun et al., 2020). Secondly, serum exo-circRNAs expression alterations were examined for the detection of alcohol dependence in a study by Liu et al. (Liu et al., 2021) which established that hsa_circ_0004771 may have a relation with the severity of alcohol dependence thus, providing novel targets for supplementary exploration on molecular mechanisms involved in alcohol dependence. Studies have been conducted profiling differentially expressed circRNAs in exosomes of patients compared to healthy individuals. Exosomal circ_DLGAP4 was documented to be elevated in exosomes isolated from diabetic kidney disease (DKD) patients compared with normal subjects. The observations indicated that exosomal circ_DLGAP4 promoted proliferation and fibrosis of MCs cells (Bai et al., 2020). In an attempt to examine if FLI1 exonic circular RNAs (FECR) functions in small cell lung cancer (SCLC), a study found that patients with SCLC with lower level of exo-FECR1 experienced longer disease remissions than those with higher exo-FECR1 level (Li et al., 2019). Another exo-circRNA, hsa_circ_0006859, that acts as a competing endogenous RNA (ceRNA) of miR-431-5p stimulated ROCK1 expression which ultimately blocked osteogenesis and encouraged adipogenesis via miR-431-5p sponging to upregulate ROCK1. This exo-circRNA thus controlled the balance between osteogenesis and adipogenesis in human mesenchymal stem cells (hBMSCs) via miRNA sponging mechanism (Zhi et al., 2021). Furthermore, exosomal circ-HIPK3 regulated miR-421/ZIC5 axis in temozolomide (TMZ)-resistant glioma thus promoting cell progression (Han et al., 2020).

### Exosomal Circular RNAs in Diagnosis

Although the biological function of exo-circRNAs remains incompletely clarified, research in their existence and manifestation levels shows possible future opportunities of discriminating patients from healthy individuals therefore, identifying novel prospective exosome-based disease biomarkers. The potential applications of exo-circRNAs as innovative disease biomarkers is determined by their expression profiles in patients compared with healthy groups. For example, circRNA transcripts were characterized from MHCC-LM3 liver cancer cells and cell derived exosomes through genome-wide RNA sequencing analyses. Exo-circRNAs were shown to be concentrated by at least 2-fold in exosomes when compared with parental cells (Li Y. et al., 2015). Provided this information, it is conceivable that exo-circRNAs can function as biological markers of various diseases to be able to support their identification. For instance, exo-circRNAs levels in colon cancer were considerably upregulated in DKs-8 cells paralleled to DLD-1 and DKO-1 cells (Dou et al., 2016). Similarly, exo-circRNA IARS expression was higher than those of control groups both in pancreatic ductal adenocarcinoma (PDAC) tissues and in plasma exosomes (Li J. et al., 2018). A total of 13 617 circRNAs were studied to discover their expression profiles in cerebrospinal fluid (CSF) exosomes from patients with immune-mediated demyelinating disease compared to the controls; the exo-circRNAs, exo-hsa_circ_0087862 and exo-hsa_circ_0012077, were identified to be highly expressed in CSF (He et al., 2019). These results indicate how exo-circRNAs have become the attention of exploration in recent years because of their remote regulatory influence and could be used as useful biomarkers in the identification and prediction of diverse diseases based on these molecules’ elevated expression levels. Similarly, upregulated expressions of circ_0067934 in laryngeal squamous cell cancer (LSCC) tissues and cells was significantly linked to tumor size, lymph node status, and distant metastasis of LSCC and had capabilities of resulting in worse survival state. Notably, circ_0067934 may function as an oncogene in this type of cancer, providing a feasible prognostic biomarker (Chu, 2020). In that regard, circ_0067934 has been depicted to be a common determinant of being a prognostic marker in patients with NSCLC and LSCC. In summary, exo-circRNAs have been clinically valuable for early diagnosis and prognosis in patients and this type of network may help in predicting potential connections of exo-circRNAs in various diseases and their target genes.

### Exosomal Circular RNAs in Treatment

Exosomes are broadly utilized as drug vesicles because of their lipid bilayer membranes that can protect and carry different cargoes including nucleic acids and small molecule drugs. A study demonstrated the successful delivery of biocompatible exosome-sheathed PSiNPs for targeted cancer chemotherapy (Yong et al., 2019), demonstrating the capability of exosome mediated chemotherapeutic delivery to enhance anti-cancer effects. Now developments in next generation sequencing have helped improve and detect circRNAs that may have a broad impact on treatment. For example, hsa_circ_0000338 upregulated in exosomes was predicted to serve as a potential target in early prediction of chemoresistance in CRC (Hon et al., 2019). Furthermore; another circRNA, ciRS-122, was positively linked with chemoresistance in CRC by acting as a miR-122 sponge, upregulating PKM2, promoting glycolysis and reducing drug susceptibility in recipient cells (Wei et al., 2020). The use of carrier systems for the delivery of therapeutic payloads to targeted cells or tissues has attracted considerable interest, and due to their ability to shuttle proteins, nucleic acids, lipids, and drugs between cells, exosomes have gained advantage in nanotherapeutics (Ortega et al., 2020). Consequently, improving and using appropriate methods and techniques is important to expose the effective use of exo-circRNAs in early prediction of treatment effectiveness. Because of their stability and high enrichment in exosomes, studies have explored the clinical value of exo-circRNAs as therapeutic targets. For instance, a drug delivery system that is exosome based with a highly applicable potential in cancer targeted therapy had been reported, in this analysis ciRS-122 could be transferred via exosomes derived from chemoresistant CRC cells to chemosensitive cells whereby glycolysis was promoted to reduce drug susceptibility in these recipient cells. This creates a basis for future clinical uses in drug-resistant CRC and proposes a novel therapeutic target through intercellular signal delivery of cirRNAs (Wang et al., 2020).

Research in the association of exo-circRNAs is leading towards the development of new methods which could significantly improve understanding of the mechanisms underlying exo-circRNAs and has the tendency to provide a new way of treatment. For instance, one of the major breakthroughs has been the introduction of biomarkers such as programmed cell death ligand-1 (PD-L1) expression and tumor mutational burden (TMB) in predicting response to immunotherapy and even though markers for monitoring treatment response are lacking, circRNAs have demonstrated an ability to regulate gene expression by targeting known miRNA targets which, amongst others include PD-L1 (Michaelidou et al., 2020). It has been reported that immunotherapy targeting PD-L1 has the potential to provide a different approach for the treatment of NSCLC (Sui et al., 2018). Exo-circRNAs can be considered for future research as noninvasive methods for identifying PD-1/PD-L1 expression from patients which can aid in the development of therapies. In cancer, antibodies that block PD-1/PD-L1 pathway could upsurge anti-tumor immunity via acting as a possible tumor suppressor therefore, regulating the reaction to anti-PD-1/PD-L1 treatments. This becoming a potential treatment target for the optimal cancer immunotherapeutic treatment (Wang et al., 2020). Exo-circRNAs have also demonstrated to function in drug resistance regulation through miRNA sponging. For instance, exosomal circ_0072083 promoted TMZ resistance via increasing Nanoghomeobox (NANOG) and administering miR-1252-5p mediated degradation and demethylation in glioma (Ding C. Y. et al., 2021) and additionally exosomal circ-XIAP stimulated DTX resistance of PC through the regulation of miR-1182/TPD52 axis further providing a promising therapeutic mark for PC chemotherapy (Zhang et al., 2021). In another study, exosomal mmu_circ_0000250 modified adipose derived mesynchymal stem cells advanced wound healing in diabetic mice by inducing miR-128-3p/SIRT1 mediated autophagy (Shi et al., 2020).

Cisplatin is used as one of the treatments of a number of cancers. In NSCLC, serum derived exosomes enhanced NSCLC cell resistance to cisplatin (DDP), cell proliferation, migration, and invasion *in vitro*; as well as tumor growth and DDP resistance *in vivo* (Xu X. L. et al., 2020). Another type of treatment used in CRC, Oxaliplatin (OXA) treatment, frequently leads to resistance (Noordhuis et al., 2019). *In vitro* and *in vivo* studies demonstrated that exosomal circ-FBXW7 led resistant cells sensitive to OXA, increased the OXA-induced apoptosis, inhibited OXA-induced epithelial-mesenchymal transition, and suppressed OXA efflux (Xu Y. Q. et al., 2021). This suggests a promising therapeutic strategy for OXA-resistant CRC patients. Since exosomal circ_0032821 has been previously reported to play part as an oncogene in GC; a study has recently been conducted to investigate its function and mechanism in OXA resistance of GC. Circ_0032821 showed high expressions in both OXA-resistant GC cells and exosomes secreted by OXA-resistant GC cells. Moreover, exosomes containing circ_0032821 secreted by OXA-resistant GC cells could boost OXA resistance, proliferation, migration, and invasion in OXA-sensitive GC cells thus, suggesting a promising therapeutic target GC treatment (Zhong et al., 2021). The identification of novel and useful exo-circRNAs to be implemented as non-invasive biomarkers is fundamental for the early discovery and treatment diseases.

## Discussions

Exosomes have been studied and shown to be of potential use as biological indicators and therapeutic approaches in different diseases through mediating the communication between cells. Even though the biological functions of exosomes are not completely clear, research in this field is being conducted focusing on the employment of these vesicles as biomarkers in disease diagnosis and management. A study published in 2020 analyzed the human MSC-derived exosomes via proteomics revealing their potential applications in different fields, to assist future researchers in selecting optimal source cells in future exosome-related studies (Wang et al., 2020). Thus, the more research is done on exosomal function, the more we will be able to discover their key purposes in disease development, progression and in maintaining health. Since the regulatory functions of circRNAs in the progression of diseases have received increasing attention, compared to other ncRNAs, circRNAs have some exclusive characteristics that include tissue-specificity, high abundance, insensitivity to exonucleases, closed loop structure and evolutionary conservation of circRNAs makes them potential targets in the diagnosis and prognosis of many diseases (Lu, 2020). Some circRNAs have been shown to be linked to the occasion, advancement and metastasis of tumors (Gao et al., 2021). In recent years, a large number of studies have identified exo-circRNAs and their functions. This review systematically summarized the functional exo-circRNAs in various diseases and a variety of exo-circRNAs that controlled disease progression in recipient cells. Importantly, the expression and active transfer of exo-circRNAs from donor to recipient cells has been found to be associated with initiation, progression, diagnosis and treatment of numerous diseases that includes various kinds of cancers, cardiovascular diseases, neurological diseases and other diseases. Therefore, their use as targets in clinical applications appears to be a promising area of future research. However, there are disadvantageous factors that contribute to the preliminary stage of research in exo-circRNAs and its slow advancement in some diseases; for example, in ND, even though it has been showed that neurons can secrete exosomes (Yu et al., 2020) there are ethical issues related to human brain biopsy being difficult to obtain, thus slowing down possible in-depth studies. In other diseases, although biological functions of certain exo-circRNAs have been recognized, there hasn’t been enough conducted studies or they are being poorly studied in general to support their proposed applications in the treatment of these diseases. For example in this review we noted only one research which identified exosomal circ_0044516 in PC (Li T. et al., 2020), showing that more studies are needed. Therefore, elucidating the mechanisms underlying exo-circRNAs still requires further investigation that will permit the delivery of specific circRNAs through exosomes to be applicable as therapeutic resolutions in the near future. An overview of the different exo-circRNAs and biological functions are summarized in Table 1. Furthermore, administering targeted, effective therapeutics to the desired tissue is amongst one of the challenges facing modern medicine. Thus, the use of exosomes as nanoparticles for the deliverance of therapeutic compartments can be beneficial in medicine at large. On another note, the analysis of the specific mechanisms through which exo-circRNAs can influence diseases would provide a deeper understanding of disease etiologies. Pursuing disease-specific exo-circRNAs and also developing new therapeutic targets is of great connotation as circRNAs have been reported to act as miRNA sponges in various diseases; exo-circRNAs have been similarly reported to promoting disease progression in diverse ways. With current instances, studying the structure of endogenous circRNAs might be implemented in designing and developing effective artificial miRNA sponges that will be applicable as regulators of disease progression. Exo-circRNAs can also show importance as targets for evaluating responses to treatment, meaning that these molecules can’t just encourage the development of diseases but can also be used in the improvement of therapies. In conclusion, since well exosomal circRNAs regulate human diseases, it is thus expected for future studies to expose their clinical use as biomarkers and targets for disease treatment.

**TABLE 1:** Roles of exo-circRNAs in various diseases and their target miRNAs.

Disease type	Exo-circRNA	Upregulation/Downregulation and biological function	Target miRNA	Ref
PC	circ_0044516	Upregulated: promoted proliferation and metastasis	miR-29a-3p	Li T. et al. (2020)
PDAC	circ-PDE8A	Upregulated: lymphatic invasion, TNM stage and poor survival rate	miR-338-MET	Li Z. H. et al. (2018)
	circular RNA IARS	Upregulated: promote tumor invasion and metastasis	miR-122	Li J. et al. (2018)
HCC	circRNA-100338	Upregulated: affected cell proliferation, angiogenesis, permeability, and VM formation ability of HUVEC, and tumor metastasis		Huang X.-Y. et al. (2020)
	circRNA_100284	Upregulated: Accelerated cell cycle and promoted proliferation	miR-217	Dai X. et al. (2018)
	circ-0051443	Upregulated: promoted cell apoptosis and arresting the cell cycle	miR-331-3p	Chen et al. (2020)
	circ-ZNF652	Upregulated: cell proliferation, migration, invasion and glycolysis	miR-29a-3p	Li Y. H. et al. (2020)
	circUHRF1	Upregulated: decreased NK cell proportion and decreased NK cell tumor infiltration	miR-449c-5p	Zhang P. F. et al. (2020)
OC	circPUM1	Upregulated: proliferation, migration, invasion and metastasis of cancer	miR-615-5p and miR-6753-5p	Guan et al. (2019)
	circFoxp1	Upregulated: cell proliferation and DDP resistance	miR-22 miR-150-3p	Luo and Gui, (2020)
	circ-0001068	Upregulated: induced PD1 expression in T cells	miR-28-5p	Wang et al. (2020)
NSCLC	circSATB2	Upregulated: proliferation, migration and invasion	miR-326	Zhang N. et al. (2020)
	circRNA_102481	Upregulated: promote cell proliferation and inhibit cell apoptosis	miR-30a-5p	Yang B. et al. (2021)
	circ_0047921	Downregulated: could distinguish NSCLC cases from COPD controls	let-7g	Xian et al. (2020)
	circ_0056285	Downregulated: associated with advanced NSCLC stages and lymph node metastasis		
	circ_0007761	Upregulated: distinguish NSCLC cases and TB controls		
	hsa_circ_0014235	Upregulated: enhanced DDP resistance and cell malignancy	miR-520a-5p	Xu X. L. et al. (2020)
	Circ-MEMO1	Upregulated: proliferation, cell cycle progression, glycolytic metabolism and inhibited apoptosis	miR-101-3p	Ding C. Z. et al. (2020)
SCLC	exo-FECR1	Upregulated: promotes tumor metastasis	miR584-3p	Li et al. (2019)
CRC	circPACRGL	Upregulated: promoted CRC cell proliferation, migration, invasion, as well as differentiation of N1 to N2 neutrophils	miR-142-3p miR-506-3p	Shang et al. (2020)
	circ-PNN/hsa_circ_0101802	Upregulated: abnormal expression may be related to CRC progression	hsa-miR-6833-3P, hsa-let-7i-3p and hsa-miR-1301-3P	Xie Y. et al. (2020)
	circ_0067835	Upregulated: knockdown repressed cell proliferation, cell cycle progression, and enhanced cell apoptosis and radiosensitivity	miR-296-5p	Wang. et al. (2021)
	circSLC7A6	Upregulated: promoter for CRC cell proliferation and invasion, inhibitor for apoptosis		Gu et al. (2020)
	circ_0000338	Upregulated: knockdown improved chemo-resistance of CRC cells78		Hon et al. (2019)
	ciRS-122	Upregulated: promoting glycolysis and drug resistance	miR-122	Wang et al. (2020)
	circ-FBXW7	Downregulated: led resistant cells sensitive to oxaliplatin, increased the oxaliplatin-induced apoptosis, inhibited oxaliplatin-induced epithelial-mesenchymal transition, and suppressed oxaliplatin efflux	miR-18b-5p	Xu Y. Q. et al. (2021)
GC	ciRS-133	Upregulated: WAT browning and play a key role in cancer-associated cachexia	miR-133	Zhang et al. (2019)
	circSHKBP1/hsa_circ_0000936	Upregulated: promoted GC cell proliferation, migration, invasion and angiogenesis	miR-582-3p	Xie M. Y. et al. (2020)
	circ-ITCH	Upregulated: inhibited the proliferation, migration, invasion and epithelial EMT of GC cells	miR-199a-5p	Wang Y. et al. (2021)
	circNRIP1	Upregulated: knockdown blocked proliferation, migration, invasion and the expression level of AKT1 in GC cells	miR-149-5p	Zhang et al. (2019)
	circ_0032821	Upregulated: boosted OXA resistance, proliferation, migration, and invasion in OXA-sensitive GC cells	miR-515-5p	Zhong et al. (2021)
Glioma	circ_0072083	Upregulated: knockdown restrained the resistance of resistant cells via decreasing IC50 of TMZ, proliferation, migration, invasion and xenograft tumor growth and increasing apoptosis	miR-1252-5p	Ding C. Y. et al. (2021)
CAD	hsa_ circ_0005540	Upregulated: distinguish patients with CAD from non-CAD controls “There is currently no definitive evidence demonstrating the biological function of hsa_circ_0005540”	miR-221 and miR-145	Wu W. P. et al. (2020)
	hsa_circ_0124644	Upregulated: Cell apoptosis, Robo receptor signaling pathway and some other cellular processes cell apoptosis		Zhao Z. Z. et al. (2017)
SZ	hsa_circ: chr3_196488683_196483770_−4913	may be involved in the pathogenesis of SZ through sponging miRNAs to regulate gene expression and/or mediating biological pathways	miR877–3p, miR1238–3p, miR1299, miR3194–3p, miR4778–3p, miR5703, miR6514–3p, miR6772–5p, miR6809–3p, miR6875–3p, miR3059–5p	(Tan et al., 2021)
	hsa_circ: chr5_143057747_143054439_+3308		miR34a5p, miR34c5p, miR449a, miR449b5p, miR767–5p, miR766–5p, miR3666, miR3192–5p, miR4738–3p, miR6827–5p	
	hsa_circ: chr6_130956499_130926605_−29894		miR518c5p, miR942–5p, miR4467, miR6851–5p, miR10400–5p, miR3085–5p	
GD	hsa_circRNA_000102	Upregulated: viral infection and interferon-beta signaling	hsa-miR-3151-5p, hsa-miR1227-5p, hsa-miR-194-3p, hsa-miR-1296-3p, hsa-miR3688-3p, hsa-miR-7112-3p, hsa-miR-8063, hsa-miR-4512 and hsa-miR-7848-3p	Sun et al. (2020)
Alcohol Dependence	hsa_circ_0004771	Upregulated: Pathogenesis and progression of AD	miR-653-5p, hsa-miR-4251, hsa-miR-8081, hsa-miR-6751-3p, and hsa-miR-339-5p	Liu et al. (2021)
DKD	circ_DLGAP4	Upregulated: promoted proliferation and fibrosis of MCs	miR-143	Bai et al. (2020)
Osteogenesis	hsa_circ_0006859	Upregulated: suppressed osteogenesis and promoted adipogenesis	miR-431-5p	Zhi et al. (2021)
Diabetes	mmu_circ_0000250	Upregulated: promoted wound healing	miR-128-3p	Shi et al. (2020)

## Abbreviations

AD, Alzheimer’s disease; circRNA, circular RNA; CCK8, counting kit 8; COPD, Chronic obstructive pulmonary disease; DDP, Ciplatin; PC, Prostate cancer; PDAC, Pancreatic ductal adenocarcinoma; HCC, Hepatocellular cancer; VM, Vasculogenic mimicry; HUVEC, Human vein endothelial cells; OC, Ovarian cancer; OSCC, Oral squamous cell carcinoma; PD-1, Programmed cell death ligand-1; NSCLC, Non-small cell lung cancer; SCLC, Small cell lung cancer; CRC, Colorectal cancer; GC, Gastric cancer; CAD, Coronary artery disease; SZ, Schizophrenia; GD, Grave’s disease; PD, Parkinson’s disease; DKD, Diabetic kidney disease; MCs, Mesangial cells; EMT, Epithelial mesenchymal transition; TMZ, Temozolomide; ECM, extracellular matrix; RBP, RNA-binding protein; OXA, Oxaliplatin.
